# IsopretGO—analysing and visualizing the functional consequences of differential splicing

**DOI:** 10.1093/nargab/lqae165

**Published:** 2024-12-05

**Authors:** Guy Karlebach, Peter Hansen, Kristin Köhler, Peter N Robinson

**Affiliations:** The Jackson Laboratory for Genomic Medicine, 10 Discovery Drive, Farmington, CT 06032, USA; The Jackson Laboratory for Genomic Medicine, 10 Discovery Drive, Farmington, CT 06032, USA; Berlin Institute of Health at Charité—Universitätsmedizin Berlin, Charitéplatz 1, 10117 Berlin, Germany; The Jackson Laboratory for Genomic Medicine, 10 Discovery Drive, Farmington, CT 06032, USA; Berlin Institute of Health at Charité—Universitätsmedizin Berlin, Charitéplatz 1, 10117 Berlin, Germany

## Abstract

Gene Ontology overrepresentation analysis (GO-ORA) is a standard approach towards characterizing salient functional characteristics of sets of differentially expressed genes (DGE) in RNA sequencing (RNA-seq) experiments. GO-ORA compares the distribution of GO annotations of the DGE to that of all genes or all expressed genes. This approach has not been available to characterize differential alternative splicing (DAS). Here, we introduce a desktop application called isopretGO for visualizing the functional implications of DGE and DAS that leverages our previously published machine-learning predictions of GO annotations for individual isoforms. We show based on an analysis of 100 RNA-seq datasets that DAS and DGE frequently have starkly different functional profiles. We present an example that shows how isopretGO can be used to identify functional shifts in RNA-seq data that can be attributed to differential splicing.

## Introduction

The advent of high-throughput RNA sequencing (RNA-seq) has brought about an exponential growth in the number of available experimental datasets from studies of differential transcription between distinct biological conditions. Extensive research efforts have been invested towards extending our capacity to interpret such datasets. To achieve this goal, it is important to be able to integrate and explore different transcriptional processes under the same analytical framework. The most common approach for characterizing the functional implications of identifying transcriptional processes associated with a condition of interest, such as treatment with a particular drug, is Gene Ontology overrepresentation analysis (GO-ORA) (1). In GO-ORA, a set of genes that are differentially transcribed under different conditions, for instance, treated versus untreated, is selected, and the overrepresentation of GO terms is assessed compared to a null model under which the genes were selected randomly. Since this procedure involves a large number of tests, it is necessary to correct for multiple testing, which is typically done by applying a false discovery rate (FDR) threshold to control the total number of false rejections. The GO-ORA approach has long been part of the standard repertoire in omics studies and has yielded many insights into biological processes that underlie certain diseases or treatments. A joint analysis of differential gene expression and alternative splicing is potentially even more informative, since each gene may display one or both of these differences, which is why the observed variances in isoform levels can originate from both (2,3). Additionally, only some of a gene’s assigned GO terms may be enriched under one condition, for example if some of the isoforms are affected by differential splicing but the gene is not differentially expressed. Therefore, a tool that allows exploring the characteristics of differential isoforms may provide a more complete understanding of the data. Currently, due to the lack of isoform-specific functional annotation, enrichment analysis tools focus on gene-level annotations and do not emphasize isoform functional differences. A number of existing works have analysed differentially spliced gene sets (4–7), but tools that specifically perform GO-ORA for differential isoforms have been lacking.

Furthermore, differential gene expression and alternative splicing are typically analysed separately as most available methods model only one of these two processes. Here, we present isopretGO, a software tool that enables researchers to explore both processes simultaneously and trace differences in isoform content to differences in domains and functions. IsopretGO uses isoform function predictions derived by our expectation–maximization algorithm (8), which we here refer to as isopretEM. IsopretGO takes as input the results of differential splicing and gene expression analysis by HBA-DEALS (2), and leverages the isoform-specific annotations of isopretEM to perform GO-ORA. IsopretGO thus provides a comprehensive overview of the functional implications of differential splicing and expression identified in bulk RNA-seq data and can thus help to improve interpretation. We demonstrate the graphical user interface (GUI) and the various analyses implemented in isopretGO using two hepatoblastoma datasets.

## Materials and methods

### Isoform-specific GO annotation predictions

We have previously presented isopretEM (isoform interpretation by EM), a method that uses expectation–maximization to infer isoform-specific functions based on the relationship between sequence and functional isoform similarity. The focus of the current work is the analysis of overrepresentation of GO terms in differentially spliced isoforms; for this, we use the predictions derived by isopretEM (8). The version of the isoform function predictions is the same as that presented in the original isopretEM publication.

### GO-ORA of differential splicing

A typical application of GO is in the analysis of lists of differentially expressed genes coming from exploratory experiments in which the transcriptional activity of all or at least most of the genes in the genome (referred to as the population set) has been measured. The basic question is whether one or more specific GO terms annotate more of the differentially expressed genes (referred to as the study set) than one would expect by chance.

A standard approach to identify the most interesting terms is to perform Fisher’s exact test for each term separately, which we here denote by GO-ORA. That is, one assesses whether the study set contains more genes annotated to a GO term *t* than would be expected by chance. For this reason, we refer to this procedure as the term-for-term approach. In order to investigate profiles of overrepresented GO terms in sets of differentially expressed genes (DGE) and sets of differentially spliced isoforms (DAS), we used HBA-DEALS to identify sets of DGE and DAS in RNA-seq datasets (2). GO-ORA of DGE is performed as described earlier. For DAS, the isoform-specific GO annotations are used. The population is defined as the set of all isoforms and their GO annotations, and the study set is defined as the set of differentially spliced isoforms as determined by HBA-DEALS.

## 100 RNA-seq datasets

For the analysis of the collection of 100 RNA-seq datasets, for each case versus control comparison, RNA-seq data were downloaded from the NCBI Sequence Read Archive (SRA) resource (9). We have selected a diverse set of conditions in order to obtain a diverse set of enriched GO terms (Supplementary Figure S1 and Supplementary Tables S1–S4). All datasets were downloaded and processed using a snakemake pipeline that performs the following steps: samples are downloaded from the SRA, quality control using fastp version 0.23.2 (10), alignment to Genome Reference Consortium Human Build 38 version 91 using STAR version 2.7.10b (11), isoform quantification by RSEM version v1.3.3 (12) and TMM normalization (13). For calling differentially expressed genes and differentially spliced isoforms, we use HBA-DEALS (2).

### Isoform-focused GO-ORA

We implemented GO-ORA with a modernized (Java 17) version of the Ontologizer software (14). The Fisher’s exact test (term for term), and parent–child union and intersection methods, which perform an analysis of GO term overrepresentation that determines overrepresentation of terms in the context of annotations to the term’s parents, and several multiple testing correction methods were implemented (14,15). For gene-level (standard) ORA, the population set is taken to be the set of all genes with at least one read in the RNA-seq experiment, and the study set is taken to be the set of differentially expressed genes. The standard, gene-level GO annotations are used for this analysis. The GO source (json) file version 2024-06-17 was used for gene-level analysis. GO-ORA is performed as described above.

### InterPro domain ORA

Using the Fisher’s exact test overrepresentation software mentioned in the previous section, an overrepresentation test is performed by defining isoforms with the presence of one or more ProSite domain annotations to be annotated to the ProSite entry. The population set is taken to be the set of all isoforms with at least one read in the RNA-seq experiment, and the study set is taken to be the set of differentially spliced isoforms. The GO annotations inferred by isopretEM are used for this analysis. Multiple testing correction was performed with the Bonferroni procedure.

### Software implementation

The algorithm described in this work was implemented as a cross-platform desktop application using the JavaFX framework. Source code, a pre-built executable and a tutorial are available at the project GitHub site (https://github.com/TheJacksonLaboratory/isopretGO). The project comprises both the GUI tool and a Java command-line application that can perform the same analyses as the GUI tool does.

### Input RNA-seq files

IsopretGO can be run starting with the output file from HBA-DEALS (2). A script for formatting the results of edgeR (16) expression and splicing analysis is also made available in the isopretGO GitHub repository (edgeR_output.R in the ‘scripts’ directory). We additionally provide a script for DEXSeq integration for differential gene expression and transcript usage analysis starting from single-cell, long-read RNA-seq data to identify and quantify transcripts expressed within single cells and cell populations. We generate pseudo-bulk expression matrices per identified cell type and use DESeq2 (17) to test for differential gene expression between conditions within each cell type. Similarly, we employ DEXSeq to test for differential transcript usage between conditions on all genes with at least two isoforms.

The DEXSeq_output.R script performs all steps from pseudo-bulking gene and transcript expression (stored in a single-cell Seurat object), transcript filtering and differential gene/isoform expression testing. Finally, it combines the test results into a format suitable for downstream analysis with isopretGO.

## Results

In this work, we present an algorithmic approach to characterize the functional implications of differential alternative splicing using GO annotations. GO-ORA is commonly performed to gain insights into differentially expressed genes significantly involved in specific biological functions or pathways (14,18), but analogous methods for examining the functional profile of differential isoforms are not yet available. A major roadblock is the lack of experimentally confirmed functional annotations of isoforms (19). We recently presented an expectation–maximization algorithm that derives GO annotations for different isoforms (8). Here, we use these predictions to perform GO-ORA on both differentially expressed genes and differentially spliced isoforms, and show that differential expression and differential splicing regulate largely distinct functional profiles. For the purposes of this work, we define ‘functional profile’ to be the set of GO terms found to be overrepresented among differentially expressed or spliced genes in an RNA-seq experiment.

### GO-ORA of differential splicing

GO-ORA has typically been used for the analysis of differentially expressed genes in RNA-seq or similar experiments, and identifies GO terms that annotate more differential genes than one would expect by chance. One can cautiously interpret overrepresentation of a GO term as a potential indication that the function represented by the GO term was affected in some way by the experiment being analysed by RNA-seq. Here, we present a method for using isoform-specific GO annotations to identify GO terms that are overrepresented in the differentially spliced isoforms (see the ‘Materials and methods’ section). Our method uses isoform-specific GO annotations that were inferred by an expectation–maximization algorithm.

### Characterization of DGE and DAS in a collection of 100 RNA-seq experiments

We assembled a collection of 100 RNA-seq experiments with a variety of focus areas, including disease pathophysiology, cancer, infectious disease, and cell and molecular biology. Sequenced reads were processed with fastp for quality control and mapped by STAR, and isoforms were quantified with RSEM (see the ‘Materials and methods’ section). DGE and DAS were called using HBA-DEALS.

**Figure 1.**
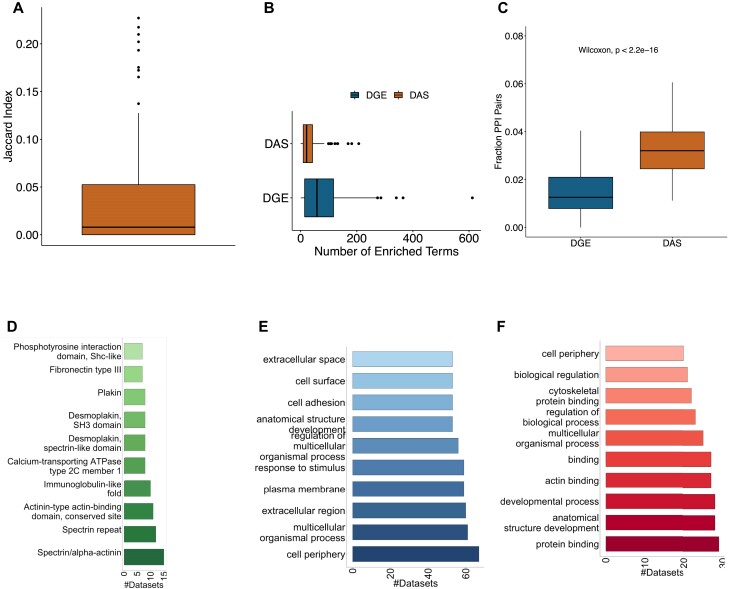
Comparison of DAS and DGE genes in 100 RNA-seq experiments. (**A**) Overlap of DGE- and DAS-enriched GO terms. The Jaccard index between GO terms enriched in DGE and DAS, computed over 100 RNA-seq datasets. (**B**) Box plots of the number of GO terms in DGE and DAS enriched per dataset. (**C**) Proportion of genes with a protein-protein interaction (PPI). The proportions of gene pairs that have a PPI in the BioGrid database, for pairs of genes that are DGE and pairs of genes that have DAS isoforms. (**D**) The most common enriched InterPro domains. (**E**) Most common DGE-enriched GO terms. (**F**) Most common DAS-enriched GO terms.

In order to assess to what extent differential expression and differential splicing are associated with the same GO terms (and thus by our hypothesis affect the same or different biological functions), we computed the Jaccard index for the sets of GO terms enriched with the sets of differentially expressed genes and differentially spliced isoforms in each dataset. Approximately 75% of all indices are <0.1, indicating that DGE and DAS are generally associated with different GO terms. Most enriched GO terms appeared as either DAS or DGE (Supplementary Figure S2). In general, there were more overrepresented GO terms for DGE than for DAS, but it is possible that this difference is related to a lower statistical power to detect differences for DAS owing to lower isoform-specific read counts or other factors (Figure 1A–C).

We additionally performed ORA for InterPro domains affected by DAS, comparing domains in differentially spliced isoforms with the population of all isoforms. The mean number of enriched domains per dataset was 21.5, and the median was 18. Co-enrichment of protein domains in the same dataset is correlated with the frequency of their co-occurrence in the same isoforms, where the former is higher than the latter (Supplementary Figure S3). This is possibly due to the fact that co-occurrence of a domain in the same isoform implies a functional relationship that can be utilized when the domains appear on different isoforms.

We calculated the proportion of gene pairs that undergo DGE and share a protein-protein interaction (PPI) and the proportion of genes that have at least one DAS isoform and share a PPI (Figure 1D; see the ‘Materials and methods’ section). The proportion was >2-fold larger for DAS (*P* = 4.4 × 10^−22^, Mann–Whitney test). This confirms and extends previously reported results on enrichment of DAS isoforms involved in PPI in cancer (20).

Several of the most common DGE-enriched GO terms were related to extracellular functions or signals, for example cell surface (GO:0009986), extracellular region (GO:0005576) and response to stimulus (GO:0050896) (Figure 1E). In contrast, several of the most common DAS-enriched GO terms were related to protein–protein interactions, for example actin binding (GO:0003779), cytoskeletal protein binding (GO:0008092), and protein binding (GO:0005515) (Figure 1F).

### GUI for exploring alternative splicing and differential expression

In contrast to other enrichment analysis tools that focus on genes, such as DAVID (21) or AmiGO (22), isopretGO is specifically designed to perform enrichment analyses at the level of isoforms and subsequently explore structural characteristics of individual isoforms found to be relevant. The GUI arranges the results in a hierarchical fashion, from summaries of differences between conditions to gene-specific and isoform-specific information. The user can navigate from the list of genes to a screen that displays the genomic organization of a gene’s isoforms, and to GO terms associated with each specific isoform. At the same time, tables that rank enriched GO terms are provided as a separate, easily accessible summary. For quick access to isoform sequence, GO and domain information, each entry is linked to external databases. IsopretGO is organized in a series of tabs.

#### Setup

The ‘Setup’ tab enables the user to download data files used by isopretGO, for example GO annotations and InterPro domains, to choose the output file of the differential analysis and to set the parameters for the GO enrichment analysis. Once these are set and the analysis is launched, isopretGO reports the outcomes in several different tabs: ‘Analysis’, ‘DGE’, ‘DAS’, ‘InterPro’ and visualization tabs for genes of interest that are labelled by the corresponding gene symbol.

#### Analysis

**Figure 2.**
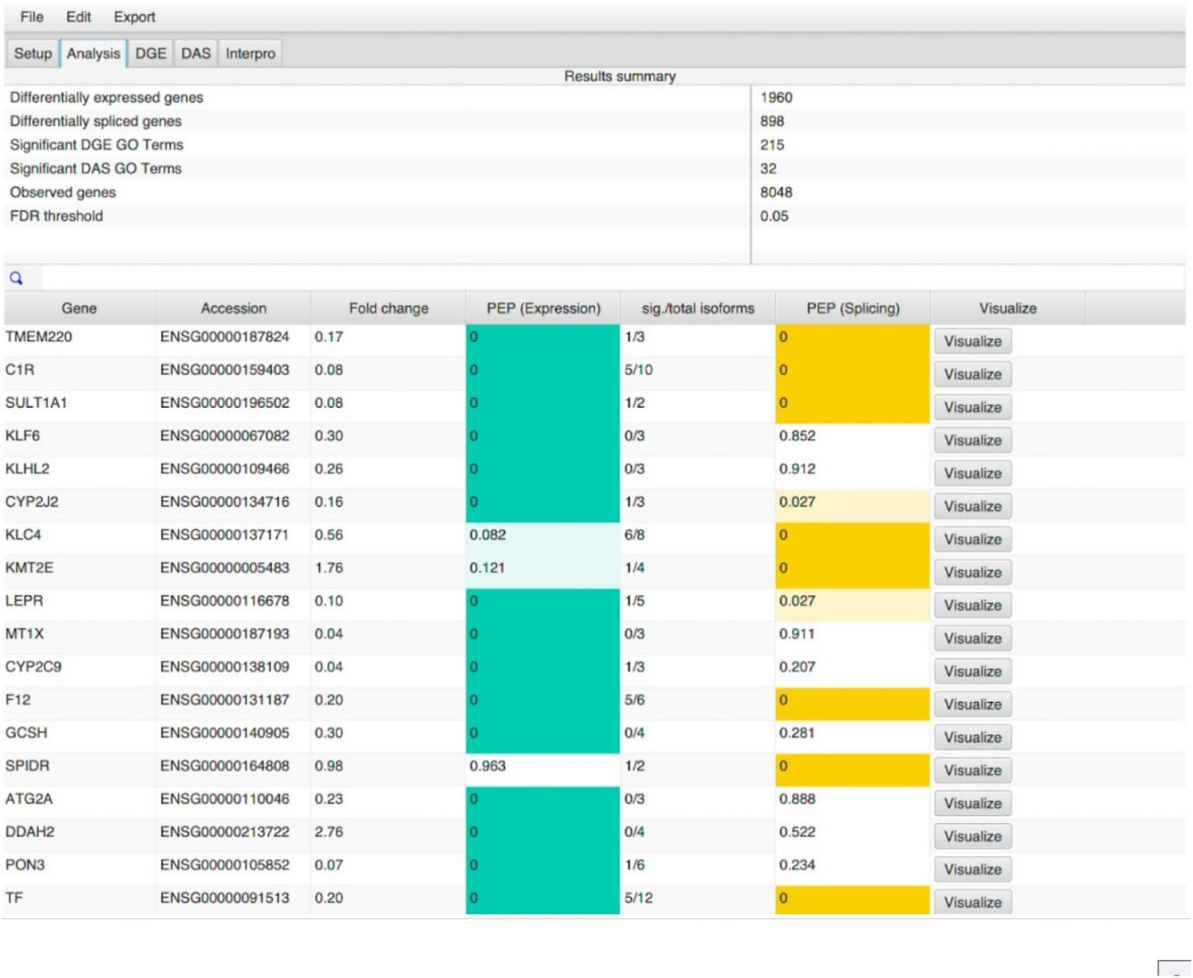
Analysis summary tab. The top frame shows the total numbers of differentially expressed genes and differentially spliced isoforms under different experimental conditions as well as the numbers of associated GO terms for which these genes are overrepresented. We define genes with at least one isoform that was assigned a high probability of proportion changes between experimental conditions as differentially expressed. In addition, the number of observed genes is shown, which corresponds to the number of genes that had at least two expressed isoforms. Finally, the FDR shown corresponds to the mean of the PEP of the smallest set of differentially expressed or differentially spliced genes that satisfies the cut-off (0.05). The bottom frame provides statistics for each gene in the dataset, and the user can visualize the structure of a gene by clicking the ‘Visualize’ button (see Figure 3). The column ‘Fold Change’ provides the log_2_ fold change of the gene expression. The column ‘PEP (Splicing)’ provides the PEP of differential splicing.

This tab displays a summary of study analysis as presented by the isopretGO GUI. The numbers of differentially spliced and differentially expressed genes are determined using the posterior error probabilities (PEPs) of differential expression and alternative splicing, where a gene is considered differentially spliced if it has differentially spliced isoforms (23). The results are shown sorted by PEP (24); by default, genes are sorted by the minimum PEP for either differential gene expression or differential splicing. By clicking on the column headers, it is possible to sort by expression or splicing PEP alone (Figure 2).

### Gene visualization tabs

**Figure 3.**
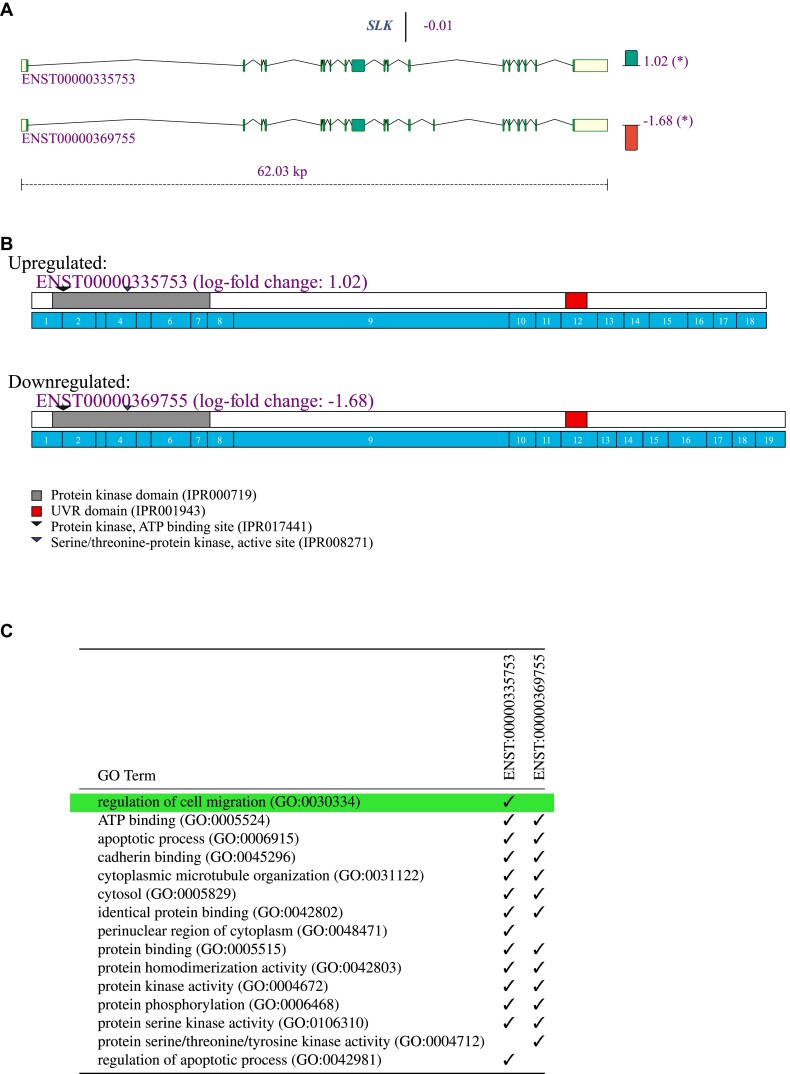
Gene visualization of the STE20-like kinase (SLK) gene. Panels (A)–(C) were exported from the isopretGO app from the gene visualization page that is opened by clicking on the ‘Visualize’ button for SLK as shown in Figure 2. (**A**) ENST00000369755.4 (isoform 1, the long isoform, 19 exons) and ENST00000335753.8 SLK-201 (isoform 2, the short isoform, 18 exons) are shown. (**B**) The protein (InterPro) domains relative to the exon structure of both isoforms are shown. (**C**) GO annotations predicted by isopretEM for both isoforms are shown. GO terms that are significantly overrepresented in the entire set of differentially spliced isoforms are highlighted.

Users can select any of the genes shown in the ‘Analysis’ tab for closer inspection by clicking on the ‘Visualize’ button in the corresponding row. This will open a new tab that is labelled by the gene symbol. The tab shows a table with the gene and its expressed isoforms, their log_2_ fold change of each isoform’s proportion between control and case samples, the log_2_ fold change of the total gene expression, and the PEP for isoform differential splicing and differential gene expression. Links to Ensembl (25) are provided for the gene and each isoform. This summary is followed by a graphical representation of the exons and introns of each isoform with respect to the gene’s genomic layout, a graphic that illustrates the location of different InterPro domains on the gene’s isoforms, and finally a table with GO terms and an indication which isoform is predicted to be annotated to each GO term is provided. GO terms that were found to be significantly overrepresented in the set of all differential isoforms across the dataset are highlighted. Speculatively, if an isoform is both differentially spliced and also annotated to a GO term that achieved statistical significance for the entire dataset, then the differential splicing event may be more likely to be biologically meaningful and worthy of follow-up. A drop-down menu is provided that allows the user to export the graphics as SVG or PDF files, or to download the GO annotations for the gene isoforms as a TSV (tab-separated value) file or a LaTeX table (Figure 3).

An example is shown for the *SLK* gene. SLK is a protein kinase that regulates apoptosis, proliferation, migration, development and tissue repair (26). Emerging evidence suggests that SLK plays important roles in cancer; for instance, loss of SLK in a murine model of HER2/Neu-positive breast cancers accelerates tumour onset and decreases overall survival (27). Figure 3 shows data on SLK derived from a hepatoblastoma dataset (28). Overall gene expression is not differential; however, one of the two isoforms is increased (ENST00000335753.8; the short isoform, 18 exons, which we will call SLK-S) and the other decreased (ENST00000369755.4; the long isoform, 19 exons, SLK-L). Both isoforms display PEPs that indicate differential splicing (Figure 3A). Inspection of the exon and domain structure shows that SLK-S has 18 exons and SLK-L has 19 exons (Figure 3B). Inspection of the table of predicted GO annotations shows that the SLK-S is predicted to be related to regulation of cell migration (GO:0030334), but the SLK-L is not annotated to this GO term (Figure 3C). A recently appeared preprint suggests that dysregulated SLK splicing impacts metastasis; SLK-L knockdown showed a significant reduction in cell proliferation, anchorage-dependent colony formation, migration and invasion (29). Although this is but one example, it displays well the intended use case for isopretGO: to find biological functions (GO terms) that display differential splicing across a dataset and to provide information about differential isoforms that may reward further follow-up.

In a second example, we use isopretGO to compare two hepatoblastoma datasets—the dataset of Hooks *et al.* (28), which provides RNA sequencing in 30 hepatoblastoma samples and matched normal liver samples from patients, and the dataset of Wagner *et al.* (30), which provides RNA sequencing of 11 control samples and 4 hepatoblastoma samples, both metastatic and non-metastatic. For comparison to Hooks *et al.*, we used the metastatic samples, since they are more likely to match in severity. Running these data through the same pipeline (see the ‘Materials and methods’ section) and using isopretGO, we identified 1960 differentially expressed genes and 898 differentially spliced genes in the Hooks *et al.* dataset, and 1401 differentially expressed genes and 1188 differentially spliced genes in the Wagner *et al.* dataset. The overlap between differentially expressed genes (986) and differentially spliced isoforms (685) in both datasets was significantly larger than expected by chance (hypergeometric *P*-value of 2.2 × 10^−308^ for both). Supplementary Figures S4 and S5 display the log-fold change correlations.

**Figure 4.**
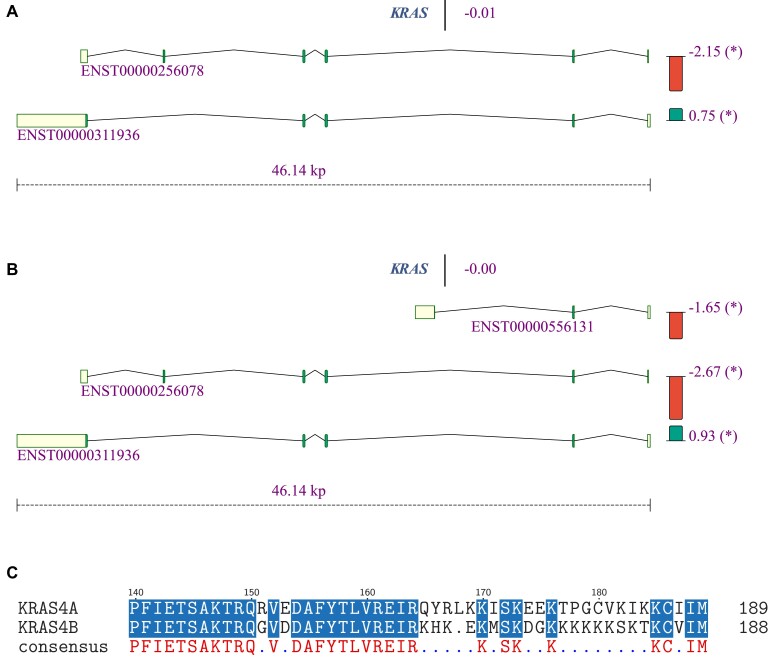
Differential splicing of the KRAS gene in hepatoblastoma. (**A**) Visualization of expressed isoforms of the KRAS gene for the data of Hooks *et al.* (28). (**B**) Visualization of expressed isoforms of the KRAS gene for the data of Wagner *et al.* (30). In both datasets, compared to the controls, isoform ENST00000256078 (KRAS4A) is downregulated, while isoform ENST00000311936 (KRAS4B) is upregulated. ENST00000556131 was observed in the second dataset. (**C**) Amino acid sequence alignment of the proteins encoded by the isoforms ENST00000256078 (KRAS4A; top) and ENST00000311936 (KRAS4B; bottom). Only the C-terminal region is shown, where the differences between these isoforms are located.

The proto-oncogene KRAS is known to undergo alternative splicing (31,32). While the gene itself is not differentially expressed in either of the datasets, its isoforms change significantly in proportions between healthy and hepatoblastoma samples—ENST00000256078 (KRAS-201, traditionally called KRAS4A, a 189-aa long protein) decreases in proportion and ENST00000311936 (KRAS-202, KRAS4B, a 188-aa long protein) increases in proportion in its stead. In (30), another shorter isoform is expressed (ENST00000556131; KRAS-203, a 43-aa protein) and decreases in proportion. In order to look for a mechanistic explanation, we aligned the protein sequences of the longer isoforms (Figure 4). The canonical RAS proteins, including KRAS, consist of two functionally distinct regions: a catalytic GTP/GDP binding domain (G-domain) and an unstructured C-terminal hypervariable region of 22–23 residues that anchor the GTPases to cellular membranes by using different combinations of membrane targeting motifs.

The last 24 and 25 amino acids in the C-terminal regions of KRAS4A and KRAS4B are coded by different exons, while the first 164 amino acids are identical. The different C-terminus results in the aforementioned difference in post-translational modification, namely the palmitoylation of KRAS4A but not KRAS4B. This is thought to result in alternative localization of KRAS4A and KRAS4B (for instance, KRAS4B but not KRAS4A is localized on lysosomes), and a number of different functional roles have been shown for the two isoforms. IsopretGO predicts the GO annotation 'homeostasis of number of cells within a tissue' (GO:0048872) for KRAS4A but not for KRAS4B (all predicted GO annotations are presented in Supplementary Table S1; none of the GO terms were significantly overrepresented in the entire dataset). Interestingly, it was shown that inhibition of KRAS4A specifically inhibits cancer stem cells (31,32). In summary, isopretGO analysis showed downregulation of KRAS4A and upregulation of KRAS4B in both hepatoblastoma datasets; since these isoforms are predicted to have different functional profiles (multiple different GO annotations), one could hypothesize that alternative splicing of these isoforms is associated with the pathobiology of hepatoblastoma (Figure 4).

#### DGE and DAS tabs

ORA follows one of several algorithms (term-for-term, parent–child union, parent–child intersection) that were implemented in the Ontologizer tool (14). We recently developed an expectation–maximization framework for predicting isoform-specific GO annotations (8). In order to perform isoform-level enrichment analysis, isopretGO uses these predictions, which includes the three sub-ontologies Biological Process, Molecular Function and Cellular Compartment. The DGE and DAS tabs show tables with GO terms and the results of ORA, which is done separately for DGE and DAS.

Examining the DAS and DGE tabs shows differential expression and differential splicing of catabolic processes such as ‘organic acid catabolic process’, ‘carboxylic acid catabolic process’ and ‘cellular amino acid catabolic process’ (summary in Table 1). These changes may reflect the metabolic reprogramming of cancer cells (33) or can correspond to a cell type-specific response. We can also compare the overlap between differential expression and differential splicing affecting pathway genes using the ‘Export’ option. For example, for the GO term ‘organic acid catabolic process’, 54 of the 110 differential genes (93%) annotated to organic acid catabolic process (GO:0016054) were both differentially expressed and differentially spliced, 52 of them were only differentially expressed and 4 were only differentially spliced.

**Table 1.** Overrepresented GO terms in two hepatoblastoma datasets

(A) Differential alternative splicing				
GO	Hooks	Hooks (FET)	Wagner	Wagner (FET)
Small molecule metabolic process (GO:0044281)	157/895 (17.5%) versus 1396/15 806 (8.8%)	4.5 $\times$^−14^	195/1183 (16.5%) versus 1654/18 943 (8.7%)	2.6 $\times$^−15^
Carboxylic acid metabolic process (GO:0019752)	99/895 (11.1%) versus 724/15 803 (4.6%)	4.73$ \times$10^−13^	129/1183 (10.9%) versus 861/18 940 (4.5%)	2.31$ \times$10^−17^
Organic acid metabolic process (GO:0006082)	101/895 (11.3%) versus 752/15 803 (4.8%)	7.72$ \times$10^−13^	135/1183 (11.4%) versus 892/18 940 (4.7%)	9.88$ \times$10^−19^
Oxoacid metabolic process (GO:0043436)	100/895 (11.2%) versus 745/15 803 (4.7%)	1.15$ \times$10^−12^	134/1183 (11.3%) versus 886/18 940 (4.7%)	1.53$ \times$10^−18^
Lipid metabolic process (GO:0006629)	110/895 (12.3%) versus 899/15 803 (5.7%)	2.47$ \times$10^−11^	138/1183 (11.7%) versus 1127/18 940 (6.0%)	4.02$ \times$10^−11^
Monocarboxylic acid metabolic process (GO:0032787)	65/895 (7.3%) versus 435/15 803 (2.8%)	1.97$ \times$10^−9^	86/1183 (7.3%) versus 501/18 940 (2.6%)	2.97$ \times$10^−14^
Carboxylic acid catabolic process (GO:0046395)	42/895 (4.7%) versus 224/15 803 (1.4%)	1.88$ \times$10^−8^	51/1183 (4.3%) versus 281/18 940 (1.5%)	2.17$ \times$10^−8^
Organic acid catabolic process (GO:0016054)	42/895 (4.7%) versus 224/15 803 (1.4%)	1.88$ \times$10^−8^	51/1183 (4.3%) versus 281/18 940 (1.5%)	2.17$ \times$10^−8^
Small molecule catabolic process (GO:0044282)	50/895 (5.6%) versus 303/15 803 (1.9%)	2.61$ \times$10^−8^	61/1183 (5.2%) versus 377/18 940 (2.0%)	3.02$ \times$10^−8^
Cellular lipid metabolic process (GO:0044255)	83/895 (9.3%) versus 690/15 803 (4.4%)	1.66$ \times$10^−7^	106/1183 (9.0%) versus 877/18 940 (4.6%)	1.51$ \times$10^−7^
**(B) Differential gene expression**				
**GO**	**Hooks**	**Hooks (FET)**	**Wagner**	**Wagner (FET)**
Small molecule metabolic process (GO:0044281)	409/1957 (20.9%) versus 848/7696 (11.0%)	9.80$ \times$10^−49^	299/1400 (21.4%) versus 956/8661 (11.0%)	1.08$ \times$10^−31^
Organic acid metabolic process (GO:0006082)	251/1957 (12.8%) versus 454/7696 (5.9%)	1.22$ \times$10^−40^	187/1400 (13.4%) versus 515/8661 (5.9%)	6.83$ \times$10^−27^
Oxoacid metabolic process (GO:0043436)	249/1957 (12.7%) versus 450/7696 (5.8%)	2.30$ \times$10^−40^	186/1400 (13.3%) versus 511/8661 (5.9%)	6.99$ \times$10^−27^
Carboxylic acid metabolic process (GO:0019752)	242/1957 (12.4%) versus 439/7696 (5.7%)	1.06$ \times$10^−38^	181/1400 (12.9%) versus 499/8661 (5.8%)	7.99$ \times$10^−26^
Small molecule catabolic process (GO:0 044 282)	130/1957 (6.6%) versus 199/7696 (2.6%)	2.86$ \times$10^−29^	99/1400 (7.1%) versus 231/8661 (2.7%)	1.01$ \times$10^−18^
Monocarboxylic acid metabolic process (GO:0032787)	154/1957 (7.9%) versus 263/7696 (3.4%)	3.52$ \times$10^−27^	118/1400 (8.4%) versus 300/8661 (3.5%)	5.16$ \times$10^−19^
Carboxylic acid catabolic process (GO:0046395)	102/1957 (5.2%) versus 144/7696 (1.9%)	8.06$ \times$10^−27^	81/1400 (5.8%) versus 167/8661 (1.9%)	5.15$ \times$10^−19^
Organic acid catabolic process (GO:0016054)	102/1957 (5.2%) versus 144/7696 (1.9%)	8.06$ \times$10^−27^	81/1400 (5.8%) versus 167/8661 (1.9%)	5.15$ \times$10^−19^
Lipid metabolic process (GO:0006629)	260/1957 (13.3%) versus 563/7696 (7.3%)	8.88$ \times$10^−25^	194/1400 (13.9%) versus 662/8661 (7.6%)	4.05$ \times$10^−15^
Oxidoreductase activity (GO:0016491)	183/1957 (9.4%) versus 356/7696 (4.6%)	3.69$ \times$10^−23^	128/1400 (9.1%) versus 396/8661 (4.6%)	1.89$ \times$10^−12^

Isopret analysis was performed as described on two RNA-seq datasets that compared hepatoblastoma to normal liver tissues. The panels show the GO terms that appeared in the top 10 annotations for both datasets. (A) For differential splicing, six terms were common to both datasets. (B) For differential gene expression, nine terms were common to both datasets. In this case, there was substantial overlap between overrepresented terms for expression and slicing, but in many cases, the overlap was much less (Supplementary Figure S2). FET: Fisher’s exact test.

The figure shows that most genes are affected by differential expression, and a subset of those are affected by differential splicing.

## Discussion

Differential expression and differential splicing both contribute to altering a gene’s transcriptional output, but can have different biological roles. Our knowledge about the contributions of alternative splicing to physiology and disease lags behind our knowledge about the roles of differential gene expression. Technology has driven discovery at least since the discovery of bacteria by Leeuwenhoek in 1677 with the relatively high-resolution microscope he developed (34). The global analysis of gene expression has been possible for roughly three decades, thanks to technologies such as gene expression microarrays, but the comprehensive and accurate investigation of alternative splicing is just beginning to be possible at scale with long-read sequencing technologies. There is therefore a substantial need for algorithms and software to support these technologies.

In this work, we introduce an extension of the traditional GO-ORA approach to investigate sets of differentially expressed genes with the Fisher’s exact test or extensions of the Fisher’s exact test. Our approach uses inferred isoform-specific GO annotations (8) and then treats each isoform analogously to a gene in the traditional Fisher’s exact test approach. Using this method, we showed that isoforms affected by differential expression and splicing are functionally disjoint over a large set of case–control studies corresponding to a broad range of conditions. We provide a desktop application called isopretGO that can be used to perform the analysis and to visualize the genes and their isoforms in the context of the protein domains affected by alternative splicing and the isoform-specific GO annotations. We present examples taken from the analysis of two hepatoblastoma datasets in which splicing and expression had complementary roles in explaining the disease condition, and show how isopretGO can be used to explore such phenomena.

A limitation of the work presented here is that although alternative splicing of messenger RNA generates numerous mature transcripts, some recent results suggest that most protein-coding genes have only a single dominant protein isoform (35–37). However, these conclusions based on proteomics studies are not universally accepted and distinguishing noise from signal in splicing remains an active research topic (38–41). Users of our software should be aware of these limitations. We speculate that a certain proportion of genes will display functionally relevant alternative splicing and suggest that the determination of which genes do so is a crucial topic for research on gene regulation.

Expression and splicing affect different biological processes both in specific responses and in contributing to different functional profiles in general. They have different effects on signalling as reflected by the propensity of their targets for protein–protein interactions, and furthermore splicing can significantly change the expression of various protein domains. IsopretGO provides sophisticated isoform-specific analysis and visualization software to support continued discovery of the roles and functions of alternative splicing in health and disease.

## Supplementary Material

lqae165_Supplemental_File

## Data Availability

No original data were generated for this work. The HBA-DEALS output files for the 100 RNA-seq experiments analysed for this project are made available at Zenodo: https://zenodo.org/records/10369086.

## Code availability

The RNA-seq pipeline is available at https://github.com/TheJacksonLaboratory/isopretGO/ in the subdirectory ‘scripts/pipeline’. It includes the Snakefile, bash files that start the execution, a configuration file and the R file that is run by the pipeline. The current version of the code used for this article has been uploaded to Zenodo: https://doi.org/10.5281/zenodo.14052970.

The isopretGO Java application is freely available under the GNU General Public License v3.0 at https://github.com/TheJacksonLaboratory/isopretGO.

## References

[1] Ashburner M. , Ball C.A., Blake J.A., Botstein D., Butler H., Cherry J.M., Davis A.P., Dolinski K., Dwight S.S., Eppig J.T. et al. Gene Ontology: tool for the unification of biology. The Gene Ontology Consortium. Nat. Genet. 2000; 25:25–29. 10802651 10.1038/75556 PMC3037419

[2] Karlebach G. , Hansen P., Veiga D.F., Steinhaus R., Danis D., Li S., Anczukow O., Robinson P.N. HBA-DEALS: accurate and simultaneous identification of differential expression and splicing using hierarchical Bayesian analysis. Genome Biol. 2020; 21:171. 32660516 10.1186/s13059-020-02072-6 PMC7358203

[3] Karlebach G. , Aronow B., Baylin S.B., Butler D., Foox J., Levy S., Meydan C., Mozsary C., Saravia-Butler A.M., Taylor D.M. et al. Betacoronavirus-specific alternate splicing. Genomics. 2022; 114:110270. 35074468 10.1016/j.ygeno.2022.110270 PMC8782732

[4] Feng H. , Li T., Zhang X. Characterization of kinase gene expression and splicing profile in prostate cancer with RNA-seq data. BMC Genomics. 2018; 19:564. 30367578 10.1186/s12864-018-4925-1 PMC6101066

[5] Li D. , Liang Y., Lu J., Tan Y. An alternative splicing signature in human Crohn’s disease. BMC Gastroenterol. 2021; 21:420. 34749666 10.1186/s12876-021-02001-2 PMC8573860

[6] Mehmood A. , Laiho A., Venäläinen M.S., McGlinchey A.J., Wang N., Elo L.L. Systematic evaluation of differential splicing tools for RNA-seq studies. Brief. Bioinform. 2020; 21:2052–2065. 31802105 10.1093/bib/bbz126 PMC7711265

[7] Lu Y. , Yue D., Xie J., Cheng L., Wang X. Ontology specific alternative splicing changes in Alzheimer’s disease. Front. Genet. 2022; 13:926049. 35774499 10.3389/fgene.2022.926049 PMC9237535

[8] Karlebach G. , Carmody L., Sundaramurthi J.C., Casiraghi E., Hansen P., Reese J., Mungall C.J., Valentini G., Robinson P.N. An expectation–maximization framework for comprehensive prediction of isoform-specific functions. Bioinformatics. 2023; 39:btad132. 36929917 10.1093/bioinformatics/btad132 PMC10079350

[9] International Nucleotide Sequence Database Collaboration Leinonen R. , Sugawara H., Shumway M. The Sequence Read Archive. Nucleic Acids Res. 2011; 39:D19–D21. 21062823 10.1093/nar/gkq1019 PMC3013647

[10] Chen S. Ultrafast one-pass FASTQ data preprocessing, quality control, and deduplication using fastp. iMeta. 2023; 2:e107. 38868435 10.1002/imt2.107 PMC10989850

[11] Dobin A. , Davis C.A., Schlesinger F., Drenkow J., Zaleski C., Jha S., Batut P., Chaisson M., Gingeras T.R. STAR: ultrafast universal RNA-seq aligner. Bioinformatics. 2013; 29:15–21. 23104886 10.1093/bioinformatics/bts635 PMC3530905

[12] Li B. , Dewey C.N. RSEM: accurate transcript quantification from RNA-seq data with or without a reference genome. BMC Bioinformatics. 2011; 12:323. 21816040 10.1186/1471-2105-12-323 PMC3163565

[13] Robinson M.D. , Oshlack A. A scaling normalization method for differential expression analysis of RNA-seq data. Genome Biol. 2010; 11:R25. 20196867 10.1186/gb-2010-11-3-r25 PMC2864565

[14] Bauer S. , Grossmann S., Vingron M., Robinson P.N. Ontologizer 2.0—a multifunctional tool for GO term enrichment analysis and data exploration. Bioinformatics. 2008; 24:1650–1651. 18511468 10.1093/bioinformatics/btn250

[15] Grossmann S. , Bauer S., Robinson P.N., Vingron M. Improved detection of overrepresentation of Gene-Ontology annotations with parent–child analysis. Bioinformatics. 2007; 23:3024–3031. 17848398 10.1093/bioinformatics/btm440

[16] Robinson M.D. , McCarthy D.J., Smyth G.K. edgeR: a Bioconductor package for differential expression analysis of digital gene expression data. Bioinformatics. 2010; 26:139–140. 19910308 10.1093/bioinformatics/btp616 PMC2796818

[17] Love M.I. , Huber W., Anders S. Moderated estimation of fold change and dispersion for RNA-seq data with DESeq2. Genome Biol. 2014; 15:550. 25516281 10.1186/s13059-014-0550-8 PMC4302049

[18] Robinson P.N. , Bauer S. Introduction to Bio-Ontologies. 2011; Boca Raton, FL, USA Chapman & Hall/CRC Press.

[19] Bhuiyan S.A. , Ly S., Phan M., Huntington B., Hogan E., Liu C.C., Liu J., Pavlidis P. Systematic evaluation of isoform function in literature reports of alternative splicing. BMC Genomics. 2018; 19:637. 30153812 10.1186/s12864-018-5013-2 PMC6114036

[20] Climente-González H. , Porta-Pardo E., Godzik A., Eyras E. The functional impact of alternative splicing in cancer. Cell Rep. 2017; 20:2215–2226. 28854369 10.1016/j.celrep.2017.08.012

[21] Sherman B.T. , Hao M., Qiu J., Jiao X., Baseler M.W., Lane H.C., Imamichi T., Chang W. DAVID: a web server for functional enrichment analysis and functional annotation of gene lists (2021 update). Nucleic Acids Res. 2022; 50:W216–W221. 35325185 10.1093/nar/gkac194 PMC9252805

[22] Carbon S. , Ireland A., Mungall C.J., Shu S., Marshall B., Lewis S. AmiGO Hub and Web Presence Working Group AmiGO: online access to ontology and annotation data. Bioinformatics. 2009; 25:288–289. 19033274 10.1093/bioinformatics/btn615 PMC2639003

[23] Kruschke J.K. Doing Bayesian Data Analysis: A Tutorial with R, JAGS, and Stan. 2015; Cambridge, MA, USA Academic Press.

[24] Käll L. , Storey J.D., MacCoss M.J., Noble W.S. Posterior error probabilities and false discovery rates: two sides of the same coin. J. Proteome Res. 2008; 7:40–44. 18052118 10.1021/pr700739d

[25] Aken B.L. , Achuthan P., Akanni W., Amode M.R., Bernsdorff F., Bhai J., Billis K., Carvalho-Silva D., Cummins C., Clapham P. et al. Ensembl 2017. Nucleic Acids Res. 2017; 45:D635–D642. 27899575 10.1093/nar/gkw1104 PMC5210575

[26] Al-Zahrani K.N. , Baron K.D., Sabourin L.A. Ste20-like kinase SLK, at the crossroads: a matter of life and death. Cell Adh. Migr. 2013; 7:1–10. 23154402 10.4161/cam.22495 PMC3544772

[27] Al-Zahrani K.N. , Abou-Hamad J., Cook D.P., Pryce B.R., Hodgins J.J., Labrèche C., Robineau-Charette P., de Souza C.T., Bell J.C., Auer R.C. et al. Loss of the Ste20-like kinase induces a basal/stem-like phenotype in HER2-positive breast cancers. Oncogene. 2020; 39:4592–4602. 32393835 10.1038/s41388-020-1315-3

[28] Hooks K.B. , Audoux J., Fazli H., Lesjean S., Ernault T., Dugot-Senant N., Leste-Lasserre T., Hagedorn M., Rousseau B., Danet C. et al. New insights into diagnosis and therapeutic options for proliferative hepatoblastoma. Hepatology. 2018; 68:89–102. 29152775 10.1002/hep.29672

[29] Yang Y.-Q. , Hu Y., Zhang S.-R., Li J.-F., Guan J.-W., Zhang W.-J., Sun Y., Feng X.-Y., Sun J., Yang Y. et al. Extensive dysregulation of SLK splicing in cancers impacts metastasis. 2023; bioRxiv doi:29 March 2023, preprint: not peer reviewed 10.1101/2022.10.28.514146.

[30] Wagner A.E. , Schwarzmayr T., Häberle B., Vokuhl C., Schmid I., von Schweinitz D., Kappler R. SP8 promotes an aggressive phenotype in hepatoblastoma via FGF8 activation. Cancers. 2020; 12:2294. 32824198 10.3390/cancers12082294 PMC7465460

[31] Rásó E. Splice variants of RAS-translational significance. Cancer Metastasis Rev. 2020; 39:1039–1049. 32772213 10.1007/s10555-020-09920-8 PMC7680328

[32] Chen W.-C. , To M.D., Westcott P.M.K., Delrosario R., Kim I.-J., Philips M., Tran Q., Bollam S.R., Goodarzi H., Bayani N. et al. Targeting KRAS4A splicing through the RBM39/DCAF15 pathway inhibits cancer stem cells. Nat. Commun. 2021; 12:4288. 34257283 10.1038/s41467-021-24498-7 PMC8277813

[33] Ward P.S. , Thompson C.B. Metabolic reprogramming: a cancer hallmark even Warburg did not anticipate. Cancer Cell. 2012; 21:297–308. 22439925 10.1016/j.ccr.2012.02.014 PMC3311998

[34] Lane N. The unseen world: reflections on Leeuwenhoek (1677) ‘concerning little animals’. Philos. Trans. R. Soc. Lond. B Biol. Sci. 2015; 370:20140344. 25750239 10.1098/rstb.2014.0344 PMC4360124

[35] Pozo F. , Martinez-Gomez L., Walsh T.A., Rodriguez J.M., Di Domenico T., Abascal F., Vazquez J., Tress M.L. Assessing the functional relevance of splice isoforms. NAR Genom. Bioinform. 2021; 3:lqab044. 34046593 10.1093/nargab/lqab044 PMC8140736

[36] Ezkurdia I. , Rodriguez J.M., Carrillo-de Santa Pau E., Vázquez J., Valencia A., Tress M.L. Most highly expressed protein-coding genes have a single dominant isoform. J. Proteome Res. 2015; 14:1880–1887. 25732134 10.1021/pr501286b PMC4768900

[37] Tress M.L. , Abascal F., Valencia A. Alternative splicing may not be the key to proteome complexity. Trends Biochem. Sci. 2017; 42:98–110. 27712956 10.1016/j.tibs.2016.08.008 PMC6526280

[38] Blencowe B.J. The relationship between alternative splicing and proteomic complexity. Trends Biochem. Sci. 2017; 42:407–408. 28483376 10.1016/j.tibs.2017.04.001

[39] Wan Y. , Larson D.R. Splicing heterogeneity: separating signal from noise. Genome Biol. 2018; 19:86. 29986741 10.1186/s13059-018-1467-4 PMC6036703

[40] Melamud E. , Moult J. Stochastic noise in splicing machinery. Nucleic Acids Res. 2009; 37:4873–4886. 19546110 10.1093/nar/gkp471 PMC2724286

[41] Bénitière F. , Necsulea A., Duret L. Random genetic drift sets an upper limit on mRNA splicing accuracy in metazoans. eLife. 2024; 13:RP93629. 38470242 10.7554/eLife.93629 PMC10932544

