# Supplementary material for: IsopretGO—analysing and visualizing the functional consequences of differential splicing
Source: NAR Genom Bioinform. 2024 Dec 5;6(4):lqae165. doi: 10.1093/nargab/lqae165 (PMC11630322; doi:10.1093/nargab/lqae165)
Supplement: lqae165_Supplemental_File [file lqae165_supplemental_file.pdf]

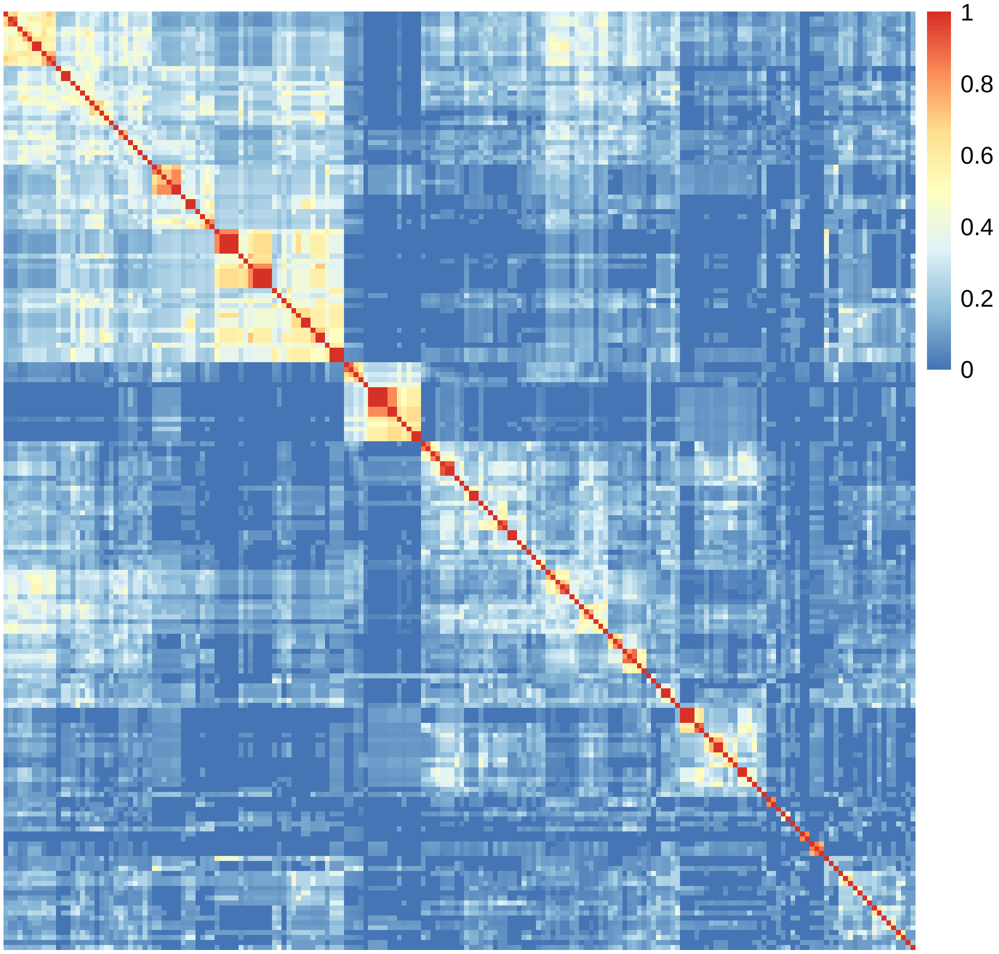

Figure **S1**: **Co-occurrence of GO terms across datasets**. The heatmap shows clusters of GO terms that co-occur as DAS-enriched in the same datasets, using our collection of 100 RNA-Seq datasets. The rows and columns correspond to GO terms that are enriched in at least 5 datasets, and the color corresponds o the Jaccard index between datasets in which they are DAS-enriched, i.e. the proportion of datasets in which both GO terms are DAS-enriched, out of all datasets that have either GO term as DAS-enriched. The figure shows that there are some clusters of datasets that are related enrichment-wise, although the overlap in DAS enrichment over all the collection is relatively low, indicating a diverse set of differential- splicing responses.

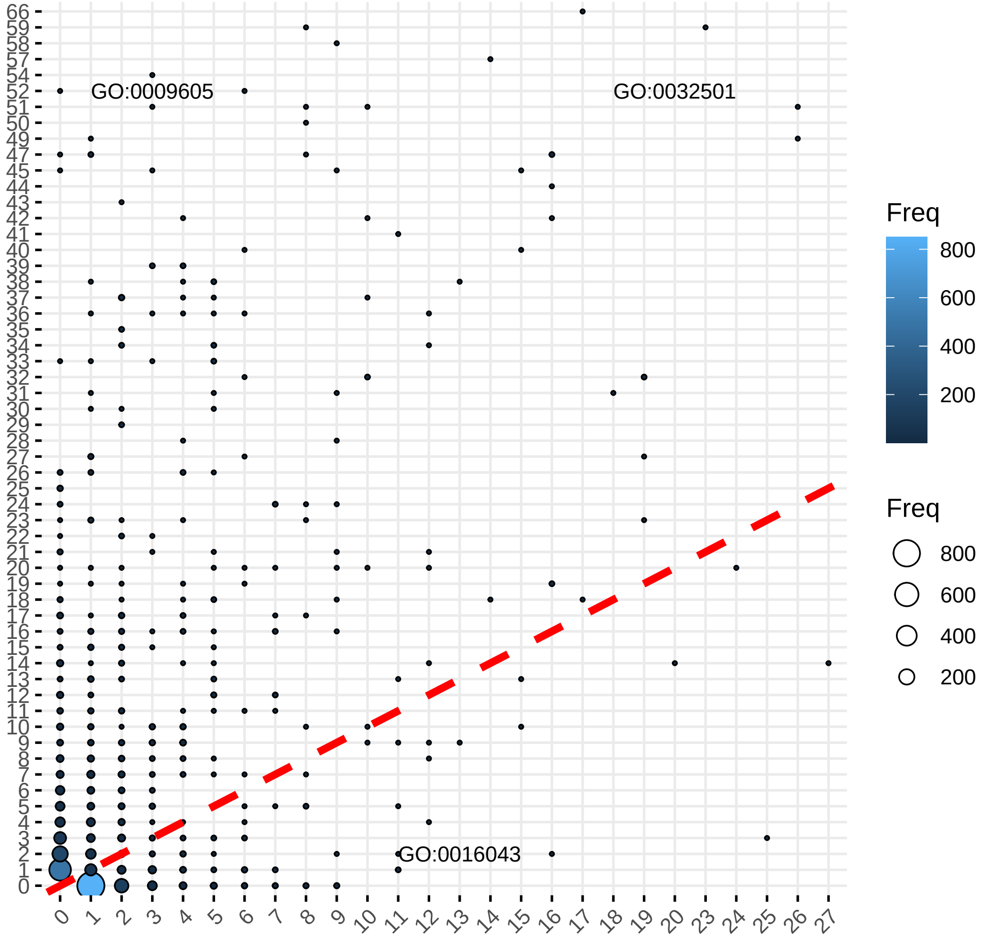

Figure **S2**: **DGE vs DAS counts for all GO terms**. The plot illustrates the number of datasets in which every GO term is DAS- and DGE-enriched, for GO terms that are enriched in at least one dataset in our collection. The x-coordinate corresponds to the number of datasets in which a GO term is enriched for DAS, the y-coordinate corresponds to the number of datasets in which a GO term is enriched for DGE, and the size of the dot corresponds to the number of GO terms for the corresponding x and y axis values apply. Some of the GO terms are displayed next to the dots that represent them. The term *multicellular organismal process* (GO:0032501) appears in as many datasets in DGE as DAS, whereas the terms *cellular component organization* (GO:0016043) and *response to external stimulus* (GO:0009605) are mostly enriched for only one of DAS or DGE.

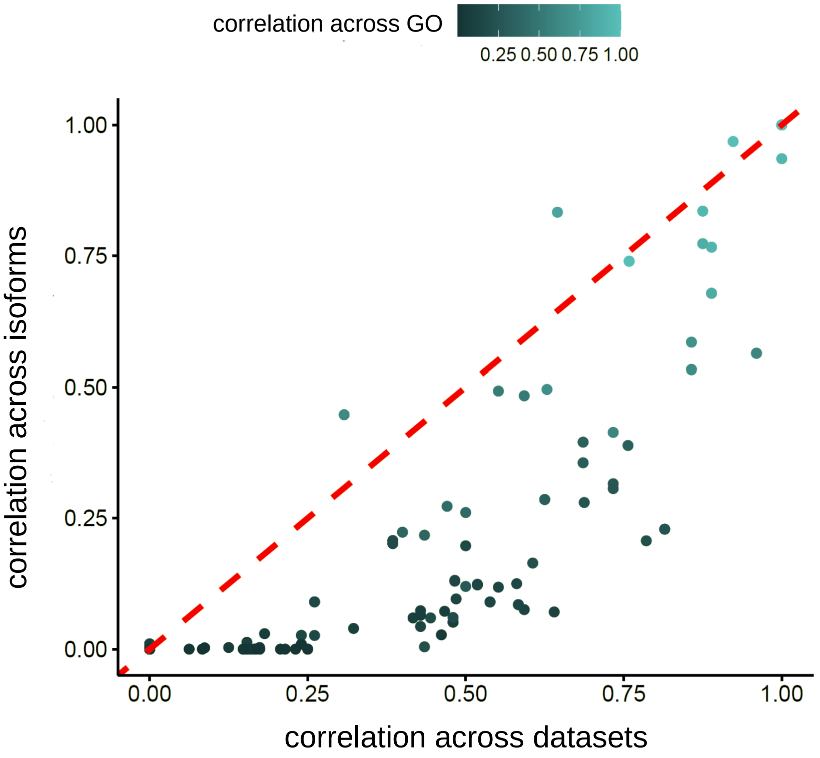

Figure **S3**: **Co-occurrence of Interpro domains in datasets and on isoforms**. The value on the x-axis of the scatter plot corresponds to the number of common datasets that a pair of Interpro domains is enriched in, divided by the total number of datasets that either of them is enriched in. The value on the y-axis corresponds to the number of transcripts that a pair of domains appears on together divided by the number of transcripts either of them appears on. Each dot on the scatter plot corresponds to one pair of Interpro domains. Only domains that are DAS-enriched in at least 10 datasets are plotted. The dashed red line is y=x. The figure shows that for domains that occur in at least 10 datasets, co-enrichment is correlated with co-occurrence (Pearson correlation 0.85), where the former is higher than the latter, likely since cooccurrence is associated with a functional relationship that can be carried out by two different isoforms, each carrying only one of the individual domains.

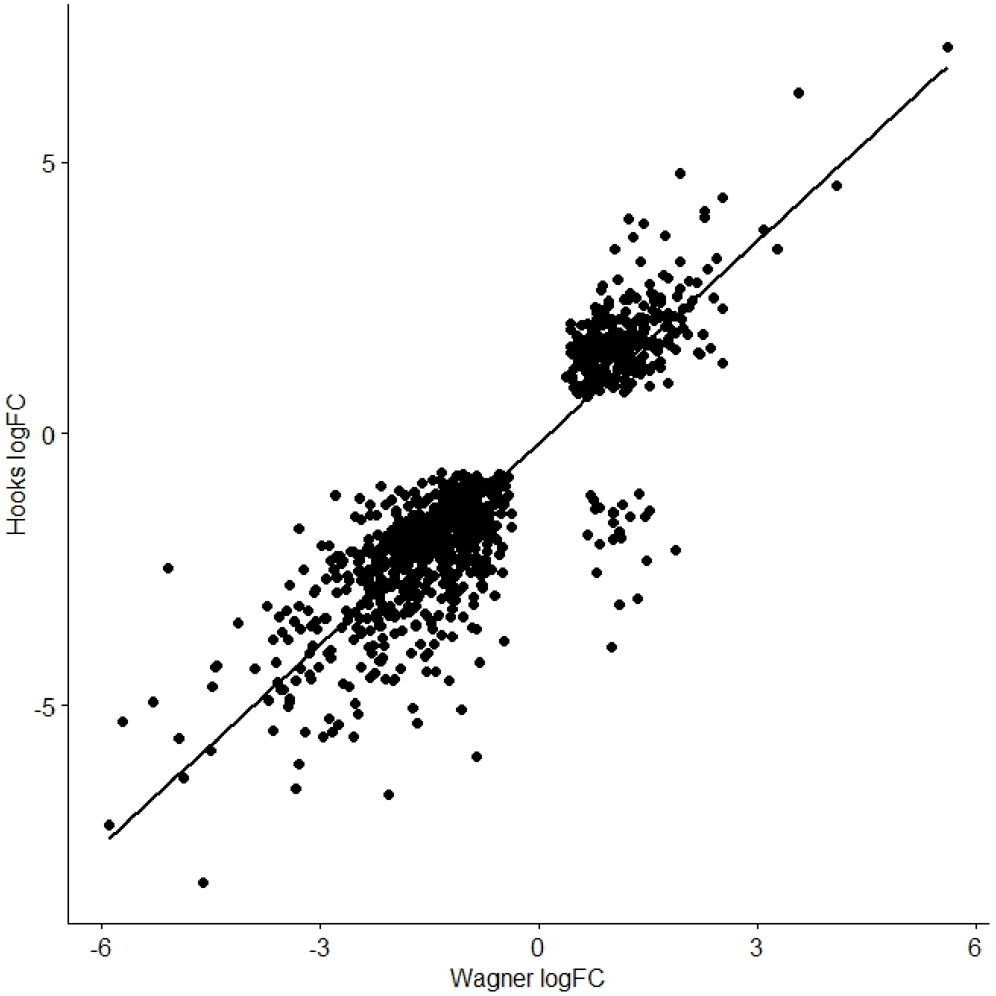

Figure **S4**: **Correlation between log fold-changes of expression levels of differentially expressed genes in the Hooks and Wagner datasets** Each dot in the scatterplot corresponds to the expression log fold-change of a gene that is included in both the Hooks (y-axis) and Wagner (x-axis) datasets' lists of differentially expressed genes. The Pearson correlation is 0.89 (p-value $< 2.225074 \cdot 10^{-308}$). The plot shows that the changes in gene expression are highly correlated between the two datasets.

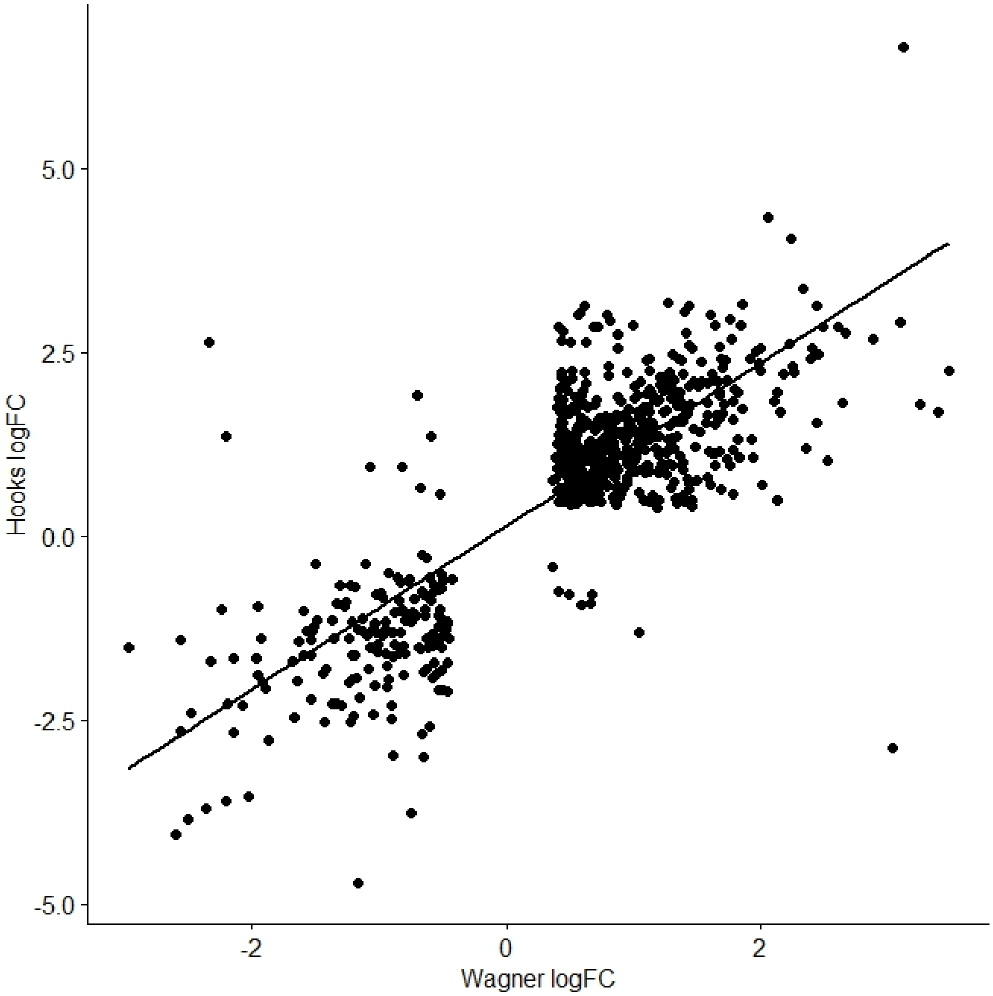

Figure **S5**: **Correlation between log fold-changes of proportions of differentially spliced isoforms in the Hooks and Wagner datasets** Each dot in the scatterplot corresponds to the log fold-change in the proportion of an isoform that is included in both the Hooks (y-axis) and Wagner (x-axis) datasets' lists of differentially spliced isoforms. The Pearson correlation is 0.81 (p-value $< 1.038621 \cdot 10^{-159}$). The proportion of an isoform depends on the levels of other expressed isoforms, but nevertheless the changes in proportions are highly correlated between the two datasets.

cohort	case:control	category	author	year	pmid
SRP120040_105	5:4	neurodegenerative disease	Osipovitch M	2019	30554964
SRP120040_106	7:4	neurodegenerative disease	Osipovitch M	2019	30554964
SRP188206_107	12:12	Neurodegenerative disease	Lavin KM	2020	31829804
SRP188206_108	12:12	Neurodegenerative disease	Lavin KM	2020	31829804
SRP051518_111	4:7	Kawasaki disease	Rowley AH	2015	26679344
SRP051518_112	4:7	Kawasaki disease	Rowley AH	2015	26679344
SRP150872_114	28:32	lupus	Davenport EE	2018	30340504
SRP332552_117	12:12	diabetes	Gabriel BM	2021	34669477
SRP223448_119	10:22	schizophrenia	Perez JM	2021	32152472
SRP325897_221	10:12	liver pathophysiology	Lv Y	2021	34413880
SRP228563_222	11:12	sickle cell disease	Creary S	2020	32487996
SRP318393_244	10:10	Sjögren	Verstappen GM	2021	34295332
SRP287301_247	8:13	enteric dysfunction.	Haberman Y	2021	33524399
SRP144583_251	18:38	enteric fever	Blohmke CJ	2019	31468702
SRP233184_261	12:12	aortic aneurysm	Chen PY	2020	32243809
SRP222046_263	4:4	abdominal aortic aneurysm	Lu H	2020	32354235
SRP219588_264	18:9	Vibrio vulnificus	Kim BS	2020	32817457
SRP279451_265	8:8	atopic dermatitis	Möbus L	2021	32615169
SRP165679_266	6:38	psoriasis	Tsoi LC	2019	30641038
SRP289501_267	9:9	asthma	Helling BA	2020	33188283
SRP234559_281	12:8	idiopathic aplastic anemia	Lim SP	2020	32294156
SRP335222_286	18:12	basophil physiology	Puan KJ	2021	34215830
SRP292025_287	6:11	neurodegenerative disease	Garofalo M	2020	33327559
SRP292025_288	6:11	neurodegenerative disease	Garofalo M	2020	33327559
SRP346219_294	4:4	peripheral vascular disease	Gross DA	2022	34793334
SRP258588_299	12:24	Burn-McKeown syndrome	Wood KA	2020	32735620
SRP262152_304	4:4	atherosclerosis	Kim JB	2020	32441123
SRP305908_306	4:4	intracerebral hemorrhage	Goods BA	2021	33749664
SRP040622_308	7:6	stroke	Huttner HB	2014	24747576
SRP328165_310	5:8	liver pathophysiology	Zhang IW	2022	34450236
SRP191103_312	8:12	gastroparesis	Herring BP	2019	31221130
SRP279451_316	21:21	atopic dermatitis	Möbus L	2021	32615169
SRP161727_321	9:5	Diamond-Blackfan anemia	Ulirsch JC	2018	30503522
SRP134188_322	8:10	dystrophic epidermolysis bullosa	Cho RJ	2018	30135250
SRP272540_323	8:6	immune thrombocytopenia	Han P	2021	33876188
SRP189352_327	4:15	SF3B1 mutations	Zhang J	2019	31474574
SRP342557_328	5:5	epilepsy	Gomes-Duarte A	2022	35310884

Table **S1**: **33 disease-related cohorts analyzed in this work**. Columms: SRP: Project (study) accession number in NCBI's Sequence Read Archive; case:control: RNA-seq sample counts for cases and controls; category: representative topic addressed by the RNA-seq experiment; author First author of the publication associated with the project; year: year of publication; pmid: PubMed identifier.

cohort	case:control	category	author	year	pmid
SRP162188_233	9:18	DRUG-seq	Ye C	2018	30333485
SRP219481_236	6:6	erythropoiesis	Rossmann MP	2021	33986176
SRP117167_242	7:7	memory T cell physiology	Belarif L	2018	30367166
SRP258620_243	7:5	macrophage physiology	Gutbier S	2020	32645954
SRP218229_249	12:8	adipose tissue	Vijay J	2020	32066997
SRP218230_253	10:6	adipose tissue	Vijay J	2020	32066997
SRP260413_255	4:6	radiation damage	Brambilla F	2020	32710624
SRP302877_269	4:4	proteasome	Cáceres-Gutiérrez RE	2022	34739170
SRP229996_271	8:4	CD8 T cell physiology	Jansen CS	2019	31827286
SRP100829_272	5:5	oocyte physiology	Reyes JM	2017	29025019
SRP202034_274	5:4	liver physiology	Mendoza A	2021	34784250
SRP224022_275	6:6	intestinal enteroids	Chang-Graham AL	2019	31029854
SRP221491_282	4:4	basal cell-like cells	Lu J	2021	32365352
SRP229589_283	4:5	tacrolimus	Dai C	2020	31941840
SRP168076_285	6:6	basophil physiology	Puan KJ	2021	34215830
SRP335222_286	18:12	basophil physiology	Puan KJ	2021	34215830
SRP094851_293	4:4	cardiomyocyte physiology	Necela BM	2017	28101782
SRP300738_295	5:5	oocyte physiology	Ntostis P	2021	34755188
SRP257383_296	4:4	development	Valcourt JR	2021	34758327
SRP344260_297	4:4	pericyte physiology	Rezaei-Lotfi S	2021	34886891
SRP253111_303	6:6	colon physiology	Bergenheim F	2020	32891909
SRP162681_307	4:4	cell cycle	Mahmoud AD	2019	31339449
SRP297875_311	6:6	gene expression	Grundman J	2021	34587152
SRP255876_313	8:8	exposure to formaldehyde	Gonzalez-Rivera JC	2020	33024153
SRP334204_317	4:4	macrophage physiology	De M	2022	35115664
SRP334204_318	4:4	macrophage physiology	De M	2022	35115664
SRP334204_319	4:4	macrophage physiology	De M	2022	35115664
SRP334204_320	4:4	macrophage physiology	De M	2022	35115664
SRP217536_324	9:10	high-protein diet	Xu C	2020	32652799
SRP149366_329	4:4	breast cells	Meng P	2019	30993572

Table **S2**: **28 RNA-seq experiments devoted primarily to the study of physiology, gene expression or cell biology**. Columms: see Table S1.

cohort	case:control	category	author	year	pmid
SRP219837_113	7:5	colorectal cancer	Orouji E	2022	34059508
SRP065445_115	7:5	Histiocytic neoplasms	Diamond EL	2016	26566875
SRP286904_223	8:8	AML	Ho JM	2020	33147339
SRP090124_224	26:11	breast cancer	Pouliot MC	2017	29108258
SRP215936_225	13:18	breast cancer	Arruabarrena-Aristorena A	2020	32888433
SRP042620_226	42:30	breast cancer	Varley KE	2014	24929677
SRP281892_227	52:26	melanoma	Hong X	2021	33203734
SRP090849_228	22:9	osteosarcoma	Scott MC	2018	29066513
SRP026537_229	7:15	breast cancer	Daemen A	2013	24176112
SRP233497_230	21:6	pancreatic cancer	Porter RL	2019	31843922
SRP331153_231	4:4	breast cancer	Arruabarrena-Aristorena A	2020	32888433
SRP092413_235	26:10	neuroblastoma	Harenza JL	2017	28350380
SRP217026_237	6:6	pancreatic cancer	Salvador-Barbero B	2020	32109375
SRP134389_238	18:4	breast cancer	Ye IC	2018	30037853
SRP247679_239	8:12	cancer	Pearson JD	2021	34270926
SRP119676_240	32:30	liver cancer	Hooks KB	2018	29152775
SRP111914_241	10:19	liver cancer	Li S	2019	30014619
SRP050440_246	7:6	resistance to BET inhibition	Rathert P	2015	26367798
SRP220467_248	8:6	retinoblastoma	Liu H	2020	33318192
SRP313282_254	9:11	lung cancer	Zhang T	2021	34493867
SRP012167_256	5:4	parathyroid adenoma	Haglund F	2012	23024189
SRP332697_257	6:6	parathyroid adenoma	Haglund F	2012	23024189
SRP332697_257	6:6	parathyroid adenoma	Haglund F	2012	23024189
SRP301216_258	5:5	colorectal cancer	Hong Q	2021	34458146
SRP327911_260	5:5	malignant pleomorphic adenoma	Han Z	2022	34986855
SRP278517_270	11:6	ovarian cancer	Cardillo N	2021	33499129
SRP303687_273	7:19	thyroid cancer	He H	2021	34238982
SRP302218_277	4:4	sarcoma	Carrabotta M	2022	34903601
SRP276412_278	5:5	GI stromal	Shao Y	2021	34458010
SRP183071_280	7:9	DLBCL	McCord R	2019	30770362
SRP311634_284	10:10	pancreatic cancer	Farshadi EA	2021	34580113
SRP183757_290	5:5	EMT-chemoresistance	Sale MJ	2019	31048689
SRP267712_291	6:6	melanoma	Grigore F	2020	32629178
SRP336449_292	5:12	head and neck cancer	Bouhaddou M	2021	34546978
SRP253895_301	6:6	cancer	Chan TW	2020	33106178
SRP253895_302	6:6	cancer	Chan TW	2020	33106178
SRP226592_305	6:6	resistance phenotypes	Johnson AG	2020	32463448
SRP312693_314	4:4	medulloblastoma	Rea J	2021	34359754
SRP254646_315	11:11	prostate cancer	He YD	2021	34301266

Table **S3**: **39 RNA-seq experiments devoted primarily to the study of cancer**. Columms: see Table S1.

cohort	case:control	category	author	year	pmid
SRP049605_116	28:13	Lyme disease	Bouquet J	2016	26873097
SRP286302_118	6:6	Trypanosoma cruzi	Gil-Jaramillo N	2021	33897690
SRP095674_220	4:6	Schistosoma haematobium	Labuda LA	2020	31844885
SRP134018_250	15:24	Tuberculous meningitis	Rohlwink UK	2019	31434901
SRP320156_309	6:6	tuberculosis	Reichmann MT	2021	34128839

Table **S4**: **5 RNA-seq experiments related to infectious disease**. Columms: see Table S1.

GO Term	ENST:00000556131	ENST:00000256078	ENST:00000311936
G protein activity (GO:0003925)	-	-	✓
GDP binding (GO:0019003)	✓	✓	✓
GTP binding (GO:0005525)	-	✓	✓
GTPase activity (GO:0003924)	✓	✓	✓
Ras protein signal transduction (GO:0007265)	-	-	✓
endoplasmic reticulum membrane (GO:0005789)	-	✓	-
epithelial tube branching involved in lung morphogenesis (GO:0060441)	✓	-	-
extrinsic component of cytoplasmic side of plasma membrane (GO:0031234)	-	✓	-
forebrain astrocyte development (GO:0021897)	✓	-	✓
homeostasis of number of cells within a tissue (GO:0048873)	-	✓	-
membrane (GO:0016020)		✓	
negative regulation of cell differentiation (GO:0045596)	-	✓	-
positive regulation of Rac protein signal transduction (GO:0035022)	✓	✓	✓
positive regulation of gene expression (GO:0010628)	-	-	✓
regulation of long-term neuronal synaptic plasticity (GO:0048169)	✓	-	✓
regulation of synaptic transmission, GABAergic (GO:0032228)	-	-	✓
signal transduction (GO:0007165)	-	✓	✓

Table **S5**: GO Annotations (isopret) for isoforms of KRAS (ENSG00000133703). ENST00000256078 (KRAS-201, traditionally called KRAS4A, a 189aa long protein); ENST00000311936 (KRAS-202, KRAS4B, a 188aa long protein); (ENST00000556131; KRAS-203, a 43aa protein).
